# State-of-the-Art Chronic Hepatitis Viruses Research in Asia

**DOI:** 10.3390/v15051172

**Published:** 2023-05-15

**Authors:** Rong-Nan Chien

**Affiliations:** 1 Division of Hepatology, Department of Hepatology and Gastroenterology, Chang Gung Memorial Hospital, Taoyuan 333423, Taiwan; ronald@adm.cgmh.org.tw; 2 Department of Medicine, College of Medicine, Chang Gung University, Taoyuan 333323, Taiwan

Approximately 400 million people worldwide are living with chronic viral hepatitis [1]. Most of them resided in the countries in the Asia-Pacific region. It is estimated that around 1 million people in this region die from chronic liver disease and its complications each year, including cirrhosis and liver cancer [2]. Chronic hepatitis B virus (HBV) and hepatitis C virus (HCV) infections cause the disease’s progression to chronic hepatitis and/or cirrhosis, and even liver cancer. Both HBV and HCV infections are also the most common cause (~78%) of primary liver cancer [3]. To prevent untoward sequelae, the diagnosis and treatment of chronically HBV- and HCV-infected patients in this region should improve.

Notably, the World Health Organization (WHO) announced a global health sector strategy for viral hepatitis and set an achievable goal to eliminate viral hepatitis B and C as public health threats by 2030 during the 69th World Health Assembly (WHA) in May 2016 [4]. These goals include achieving a 90% diagnosis rate for HBV- and HCV-infected population, an 80% treatment rate for those who need treatment, and reducing the 65% mortality rate caused by hepatitis viruses’ infections.

Regarding the research on HBV, many studies exploring the immunopathogenesis and outcomes of chronic HBV infection in recent years have provided a better understanding of its history [5,6]. Molecular biology research further explores HBV-related carcinogenesis [7]. Furthermore, all the international guidelines for HBV treatment suggest that pegylated interferon alfa (Peg-IFN-α), entecavir (ETV), tenofovir disoproxil fumarate (TDF), and tenofovir alafenamide (TAF) are the first-line therapies for chronic hepatitis B [8]. Although Peg-IFN-α is no longer on the market, a novel pegylated interferon (ropeginterferon alfa-2b), extra-long-acting interferon has shown high efficacy and a favorable safety profile in treating chronic viral hepatitis [9]. In addition, a global paradigm shift from indefinite long-term therapy to finite nucleos(t)ide analogue (NUC) therapy in hepatitis B e antigen (HBeAg)-negative patients has been emerging [6]. Many studies have shown that finite NUCs therapy may increase the off-NUC HBsAg loss (functional cure) [6]. In addition, several new drugs used to achieve the functional cure, which target different steps of the HBV life cycle, such as entry inhibitors, HBsAg excretion blockers, capsid inhibitors, and targeting cccDNAs, and improve the host’s innate and adaptive immunity are highly anticipated [10].

Since the transmission routes of HBV, HCV, and hepatitis delta virus (HDV) are quite similar, either through the blood or body fluids, it is not uncommon to encounter dual viral infection of HBV + HCV or HBV + HDV during daily practice. It is estimated the number of individuals with the HBV + HCV co-infection is approximately 2.5–10.2 million, and approximately 2.6–5.1 million worldwide have the HBV + HDV dual infection [8]. Upon dual viral infection, the influence of liver stiffness regression [11] and dynamic changes in HBV and HCV [12] after antiviral therapy are explored in this issue. The novel therapeutic consideration of inhibiting the entry of HBV+HDV has also been recently studied [13].

Regarding the research on HCV, the recent availability of pan-genotypic direct-acting antivirals (DAAs) used to treat chronic HCV infections can achieve a sustained virologic response (SVR) rate of 98–99% after a 8–12-week treatment. Although pan-genotypic DAAs showing the highest SVR rate, without any national policies or strategies, a lot of work be conducted to achieve HCV elimination by 2030 is impossible. These include enforcing policies, financing a national program, implementing a harm-reduction program, expanding the treatment capacity, removing treatment restrictions, monitoring and evaluating patients, and designing an awareness and national screening program and a national linkage-to-care program [14]. More importantly, almost 50% of patients infected with HCV are unaware in most countries, including Taiwan [14]. Hence, implementing a nationwide screening program is the way to identify the undiagnosed HCV-infected patients. In this issue, Huang et al. reported a community-based screening program using two quantitative antigens to identify both hepatitis B and C infectivity [15] and demonstrated its feasibility as a screening tool.

Finally, I hope all countries in the Asia-Pacific region will accelerate the speed of the elimination of hepatitis virus by 2030 and achieve the goal set by the WHO.
